# Financial reimbursement incentives in the use of biosimilars for rheumatoid arthritis in Japan

**DOI:** 10.1080/20523211.2026.2633832

**Published:** 2026-02-25

**Authors:** Hiroaki Mamiya, Toshiki Fukasawa, Koji Kawakami

**Affiliations:** a College of Pharmaceutical Sciences, Ritsumeikan University, Kusatsu, Japan; b Department of Pharmacoepidemiology, Graduate School of Medicine and Public Health, Kyoto University, Kyoto, Japan; c Department of Digital Health and Epidemiology, Graduate School of Medicine and Public Health, Kyoto University, Kyoto, Japan

**Keywords:** Reimbursement policy, Biosimilar, Infliximab, Etanercept, Interrupted time-series analysis

## Abstract

**Background:**

Biosimilars present a significant opportunity for cost savings. However, the uptake of biosimilars has been inconsistent across different regions and drugs, highlighting the need for effective policy interventions. This study aimed to investigate the impact of Japan's reimbursement incentive policy on the utilisation of etanercept and infliximab biosimilars among patients with rheumatoid arthritis.

**Methods:**

We conducted an interrupted time-series (ITS) analysis using data extracted from the JMDC claims database in Japan. Participants included those prescribed either the brand-name biologics or their biosimilars. The primary outcome was the proportional use of biosimilars relative to the total use of both biosimilars and originator drugs.

**Results:**

The ITS analysis demonstrated varied responses to the reimbursement policy across the two biosimilars. For infliximab, although the policy did not result in a significant level change (0.14%; 95% confidence interval [CI]: −2.83, 3.11), there was a positive but nonsignificant slope change of 0.21% per month (95% CI: −0.13, 0.55). In contrast, for etanercept, the policy led to a significant level change, with an immediate increase in use by 13.48% (95% CI: 7.82, 19.14). However, the slope change showed a significant decrease by −1.09% per month (95% CI: −1.50, −0.68).

**Conclusion:**

The results indicate that while the reimbursement policy was associated with a short-term increase in the uptake of etanercept biosimilars, it had limited impact on infliximab biosimilars. This variation suggests that financial incentives alone may not be sufficient to enhance biosimilar adoption and that policies must consider drug-specific and healthcare setting-specific factors.

## Background

Biologics are the major driver of drug spending globally, and are expected to reach 892 billion dollars by 2028, about 39% of global drug spending (IQVIA, 2024). Biosimilars, which are highly similar products to originators in terms of structure, function, quality, and clinical efficacy and safety, have huge potential for savings in drug spending (Declerck et al., 2017; IQVIA, 2024; Lyman et al., 2018; Mulcahy et al., 2022). To promote the uptake of biosimilars, many countries, especially European countries, have introduced several incentives and policies with regard to pricing systems, prescribing requirements, substitution, and supply-side measures (Böhm et al., 2023; Moorkens et al., 2017; Renwick et al., 2016; Rémuzat et al., 2017; Vogler et al., 2021). Compared to these European countries, however, biosimilar uptake in some countries has been low, including the US and Japan (Matsumoto et al., 2021; Qureshi et al., 2019; Yu et al., 2023).

In Japan, the government sets the reimbursement price paid for drugs by the National Health Insurance. At market entry, all biosimilars are set at a 30% discount to the official price of the respective originator (Mamiya & Igarashi, 2021; Qureshi et al., 2019). Unlike some European countries, Japan does not impose measures regarding physician prescribing requirements or substitutions in pharmacies, and the price of biosimilars is therefore one of only a few incentives. While biosimilars offer advantages to hospitals when fully covered for in-hospital use under the Diagnosis Procedure Combination (DPC) system – a Japanese inpatient payment system that determines reimbursement based on diagnosis and treatment procedure groups – utilisation has remained low for drugs prescribed in outpatient settings, even though infliximab biosimilars have been approved since 2014 (Kuribayashi et al., 2023). Therefore, to incentivize biosimilar uptake at medical institutions, the Japanese government introduced a new premium, namely an add-on to reimbursement fees, under the medical fee schedule as part of 2020s reimbursement policy reform. This premium is set at 1,500 yen (about 10 US dollars, based on an approximate exchange rate of 150 yen to 1 US dollar), and medical institutions can claim the premium up to three months after prescription of a biosimilar by a staff physician, for both new start and switching (Ministry of Health, Labour, & Welfare, 2020).

The effectiveness of this add-on incentive policy to reimbursement for biosimilars will be critical to whether it is worth the limited budget and whether it should be continued in Japan. In addition, we speculate that the simplicity and generalisability of this policy will make it a good case study for policy makers in other countries, since add-on incentives to reimbursement have been found in the past in Europe for generics, and direct financial incentives for clinical units have been examined in France for biosimilars (Kanavos et al., 2008; Tano et al., 2023). Moreover, since biosimilars lack some of the original drug's indications, analysis within the same indication rather than by overall drug sales simplifies the evaluation of the policy's direct effects. Therefore, we conducted an interrupted time-series (ITS) analysis to examine the effect of reimbursement incentive policy on the uptake of the biosimilars etanercept and infliximab for rheumatoid arthritis in Japan.

## Methods

### Data source

Data were obtained from the JMDC claims database (JMDC Inc.), which collects information from multiple health insurance societies and as of March 2023 covers 16 million company employees and their family members under 75 years old in Japan (Nagai et al., 2021). The database contains information on demographics, diagnoses, drug prescriptions, and procedures, and has been extensively used in epidemiological studies (Mamiya et al., 2025; Yoshida et al., 2022). The dataset for the present study spans from 1 January 2005 to 31 March 2023.

### Samples

Individuals aged 18 to 74 years who had been diagnosed with rheumatoid arthritis and prescribed either brand-name products or biosimilars were identified. For infliximab, prescriptions were included from the date of launch of its biosimilar, on 1 December 2014 to 31 March 2023; and for etanercept, prescriptions were included from the date of launch of its biosimilar, on 1 June 2018, through 31 March 2023. Eligibility required that each prescription be accompanied by an ICD-10 rheumatoid-arthritis diagnosis code in the same claim month. This claims-based case definition has been validated in Japan and shown to yield a positive predictive value of approximately 86% for rheumatoid arthritis (Kubota et al., 2021). For the infliximab analysis, individuals with claims for Behçet’s disease or Kawasaki disease in the same month as the prescription were excluded to eliminate indications held only by the originator (brand name product) and thereby ensure that the originator and its biosimilar were assessed under the same clinical indications. Individuals prescribed both the originator and its biosimilar in the same month were excluded. A code list of the inclusion and exclusion criteria is provided in eTable 1 in Supplemental Material.

### Policy of interest

In Japan, a new premium – an add-on to reimbursement fees at 1,500 yen (about 10 US dollars) – was introduced for biosimilars for both new start and switching under the medical fee schedule. Medical institutions can claim the premium up to three months after the prescription of a biosimilar by a staff physician. The policy was implemented for biosimilars for etanercept from April 2020, and to biosimilars for infliximab from April 2022 (Ministry of Health, Labour, & Welfare, 2020, 2022b). The time after policy implementation for each biosimilar was defined as an exposure. No other financial incentives for new start or switching policies for biosimilars were implemented before or after this policy. Also, because the biosimilar add-on fee can legally be claimed only for prescriptions dated on or after 1 April 2020 or 1 April 2022, physicians had no financial incentive to modify their prescribing behaviour in advance of the policy change.

### Outcomes

The outcome was defined as the proportional use of biosimilars to total originator and biosimilar use for each of etanercept and infliximab, using all claims per month.

### Statistical methods

We conducted an ITS analysis, a robust quasi-experimental design that can control for secular trends present in health system outcomes (Bernal et al., 2017; Hategeka et al., 2020; Tugwell et al., 2017). We selected interrupted time series as our primary quasi-experimental design due to the absence of a comparable, unexposed control group during our observation period. This approach is consistent with methodological guidance for evaluating discrete policy interventions when outcome data are collected at regular intervals. The repeated outcomes over time before and after the implementation of a policy were measured and the changes in the level (intercept) and trend (slope) before and after the policy intervention periods were compared to evaluate the effectiveness of the policy. We modelled the level and trend change by ITS segmented linear regression analysis in the proportional uptake of biosimilars for etanercept and infliximab after policy intervention. To assess the robustness of our findings against different model specifications for autocorrelation, we conducted sensitivity analyses by re-estimating the ITS models using no autocorrelation adjustment, as well as first-order (AR (1), primary model), second-order (AR (2)), and third-order (AR (3)) autoregressive error models. Data preparation was conducted using SAS 9.4 software (SAS Institute Inc., NC, USA), and analysis was performed with STATA 18.0 (StataCorp LLC, Texas, USA).

### Ethics approval

As all data used in the study were anonymised, informed consent from patients was not required. This study was approved by the Ethics Committee of Kyoto University Graduate School and Faculty of Medicine (no. R3979).

## Results

### Samples

We identified 24,774 claims for infliximab and 70,186 claims for etanercept for a diagnosis of rheumatoid arthritis, including both brand-name products and biosimilars. For infliximab, the total number of claims recorded during the pre-intervention period from December 2014 to March 2022 was 21,717, while 3,057 in the post-intervention period from April 2022 to March 2023. For etanercept, the number of claims was 22,922 in the pre-intervention period from June 2018 to March 2020, and 47,264 in the post-intervention period from April 2020 to March 2023. By sex, 67% of claims for infliximab in the pre-intervention period and 62% in the post-intervention period were for female patients, as were 85% of claims for etanercept in the pre-intervention period and 84% in the post-intervention period. By age, the age groups of 50–69 years accounted for 57% of pre-intervention and 64% of post-intervention claims for infliximab, and 54% and 56% for etanercept, respectively (Table 1).

**Table 1.** Sample characteristics.

Characteristic	Infliximab		Etanercept	
	Pre-intervention period (Dec. 2014–Mar. 2022)	Post-intervention period (Apr. 2022–Mar. 2023)	Pre-intervention period (Jun. 2018–Mar. 2020)	Post-intervention period (Apr. 2020–Mar. 2023)
Total claims, No.	21,717	3,057	22,922	47,264
Sex				
Female	67%	62%	85%	84%
Male	33%	38%	15%	16%
Age group, years				
18–29	4%	3%	3%	3%
30–49	36%	29%	40%	37%
50–69	57%	64%	54%	56%
≥70	3%	4%	3%	4%

Additionally, we plotted the monthly prescription counts of originator and biosimilar products for each drug separately (Supplemental Figure S1A and S1B). The infliximab series showed no marked decline after April 2020, the month when the etanercept incentive was introduced.

### Interrupted time-series analysis

**Figure 1.**
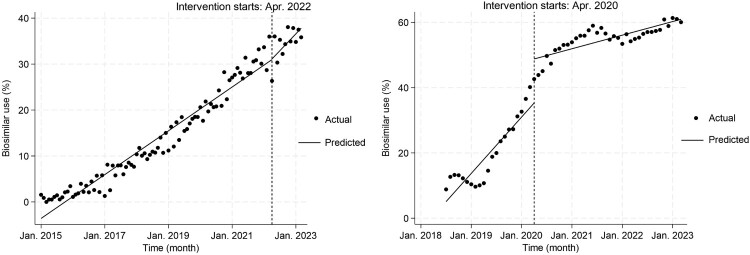
Interrupted time-series analysis of proportional use of biosimilar infliximab (**A**) and etanercept (**B**).

Table 2 displays coefficients from ITS analysis for both infliximab and etanercept. For infliximab, the pre-intervention slope showed a gradual increase at a rate of 0.40% per month (95% confidence interval [CI]: 0.37, 0.42), versus a modest level change after the policy intervention of 0.14% (95% CI: −2.83, 3.11). The post-intervention change in slope was positive, indicating an accelerated monthly increase of 0.21% (95% CI: −0.13, 0.55). This post-intervention slope suggests a continued but slight increase in the use of infliximab biosimilars at a rate of 0.61% per month (95% CI: 0.27, 0.95) (Figure 1(A)).

**Table 2.** Interrupted time-series estimates of the proportional use of biosimilars (% change).

Monthly % change (95% CI)	Infliximab		Etanercept	
Pre-intervention slope	0.40	(0.37, 0.42)	1.44	(1.03, 1.85)
Level change	0.14	(−2.83, 3.11)	13.48	(7.82, 19.14)
Slope change	0.21	(−0.13, 0.55)	−1.09	(−1.50, −0.68)
Post-intervention slope	0.61	(0.27, 0.95)	0.35	(0.23, 0.47)

Abbreviation: CI, confidence interval.

In contrast to that for infliximab, the pre-intervention slope for etanercept was considerably steeper, at 1.44% per month (95% CI: 1.03, 1.85). Following the intervention, there was a significant level change, with a substantial increase of 13.48% (95% CI: 7.82, 19.14). Conversely, the post-intervention period for etanercept showed a significant decrease in growth rate, with a slope change of −1.09% per month (95% CI: −1.50, −0.68). Despite this reduction, the post-intervention slope remained positive, albeit at a reduced rate of 0.35% per month (95% CI: 0.23, 0.47) (Figure 1(B)).

Sensitivity analyses using different autocorrelation adjustment specifications (no adjustment, and AR(1), AR(2), and AR(3) models) yielded consistent estimates for both the level and slope changes for infliximab and etanercept (eTable 2 in Supplemental Material).

## Discussion

Our study indicates that the reimbursement incentive policy was associated with a short-term increase in the uptake of etanercept biosimilars in Japan, but did not show a statistically significant change in the use of infliximab biosimilars. For infliximab, no significant level or slope changes were attributable to the policy, but its use seems to be on the increase (Table 2, Figure 1(A)). However, given that the post-intervention period in our data extends only 12 months after policy implementation, these estimates should be regarded as preliminary with respect to long-term effects. Conclusive measurement of the policy's impact on infliximab requires further investigation with extended follow-up data. Conversely, etanercept showed a significant initial uptake, which was subsequently followed by a reduction in growth rate (Table 2, Figure 1(B)). Several explanations may account for this pattern. First, the pre-intervention slope for etanercept was already steep (1.44% per month), suggesting that biosimilar adoption was already gaining momentum before the policy was implemented. The large level change may therefore represent an acceleration of adoption that was already imminent, rather than the initiation of entirely new prescribing behaviour. Second, the subsequent decline in slope is consistent with a ceiling effect, wherein the pool of physicians and patients willing to switch was partially exhausted in the initial months following the incentive. Third, the structure of the incentive itself, which limits reimbursement to three claims per patient, may have reduced the ongoing financial motivation for continued switching after the initial period. These patterns highlight the complexity of the biosimilar market, suggesting that simple measures such as economic rewards will not increase penetration; rather, policies need to be dynamically adjusted to the characteristics of the individual drug and the healthcare environment.

One reason for the differential impact of the policy on infliximab and etanercept biosimilars may be attributable to the variation in the range of approved indications between the originator products and their biosimilars. Infliximab biosimilars, for instance, do not cover certain indications such as Behçet’s disease, even though these are covered by the originator. This limitation likely influences hospital formulary decisions, favouring the originator when a choice between the two is necessary due to the broader spectrum of approved uses. For etanercept, in contrast, indications for the originator and its biosimilars are almost identical, which does not restrict its adoption in clinical settings. A second reason for the differential uptake may be related to modes of administration and dosing schedules. Infliximab is administered intravenously, typically every eight weeks during the maintenance phase, whereas etanercept is administered via subcutaneous injection once weekly. This difference implies that, if concerns arise regarding the efficacy or safety of etanercept biosimilars, transition back to the originator is relatively straightforward, providing reassurance to healthcare providers and patients. This ease of switching may have contributed to a more rapid adoption of etanercept biosimilars compared to infliximab. Furthermore, infliximab is frequently prescribed for rheumatoid arthritis in outpatient settings, suggesting that, in the context of the comprehensive care environment where the incentive is applicable, it exhibits minimal differences compared to etanercept, which is also administered in outpatient settings. Importantly, however, etanercept can be self-administered, and the intravenous administration route itself imposes an operational burden on healthcare facilities compared to self-administration. The operational costs and complexities associated with intravenous drugs may have diminished the perceived value of the relatively modest 1,500-yen incentive for infliximab biosimilars. Consequently, the policy's impact on infliximab uptake was relatively negligible compared to that on etanercept, which requires fewer resources for administration due to its self-injectable formulation.

Our study's findings present some inconsistencies with existing research. For example, in France, direct financial incentives to clinical units have significantly contributed to increasing the prescription rates of biosimilars (Tano et al., 2023). The difference in the monetary amount of these rewards in Japan and France is considered significant, and a likely cause of the difference between the countries: healthcare institutions in France are redistributed 20−30% of biosimilar sales revenues, versus only about 1500 yen (10 US dollars) in Japan. The 1,500-yen add-on is modest and can be offset by strategic responses from originator manufacturers. These tactics may include offering discounts based on unit price, providing hospitals with volume-based rebates, or intensifying detailed efforts targeting prescribers. Such contracting and discounting practices may offset the relatively modest 1,500-yen incentive in some settings; however, because these arrangements are typically confidential, we cannot directly measure them in claims data and therefore present them here as plausible mechanisms consistent with prior policy reviews (Kanavos et al., 2008).

The differential impact observed between infliximab and etanercept could reflect variations in market maturity, the number of biosimilar entrants, the remaining patent life for non-rheumatoid arthritis indications, and the strategic importance of each molecule to the originator. For example, if the infliximab market were deemed more critical, more commercial resources might be allocated to maintain market share. These contractual arrangements and commercial strategies are usually confidential, and our claims data cannot quantify them or their impact directly. We therefore present these explanations as untested hypotheses in the present study. However, the possibility of such competitive behaviour highlights the importance of considering these market dynamics when designing and evaluating financial incentives for biosimilar uptake. If substantial premiums cannot be allocated for incentives to the medical institution, it may be more effective to implement policies that encourage switching (McClean et al., 2023).

Our findings carry several implications for the design of biosimilar promotion policies. First, the contrast between the French experience, where healthcare institutions receive 20–30% of biosimilar sales revenue, and Japan’s modest 1,500-yen add-on suggests that the magnitude of financial incentives matters substantially. However, increasing the incentive amount must be weighed against budgetary constraints. Second, the differential response between etanercept and infliximab indicates that a uniform incentive may be insufficient for drugs with complex administration requirements or divergent indication profiles. A tiered incentive structure that accounts for drug-specific characteristics may be more effective than a flat-rate approach. Third, our results support the view that demand-side financial incentives alone are unlikely to achieve high biosimilar penetration rates; combining them with complementary measures such as mandatory switching policies or formulary-level preferred product designations may yield more durable effects.

In terms of the savings in drug costs by biosimilar use, the analysis indicates a significant reduction with respect to etanercept. As of April 2020, when the policy was introduced, the price of brand-name etanercept and its biosimilars for 50 mg in a pre-filled pen was 25,171 yen (about 168 US dollars) and 17,025 yen (about 114 US dollars), respectively (Ministry of Health, Labour, & Welfare, 2022a). According to Japan's National Database (NDB), the volume of original etanercept prescriptions for outpatients in 2019 was 913,779 (Ministry of Health, Labour, & Welfare, 2021), so assuming a 13% replacement with the introduction of the policy (Table 2), a reduction in drug costs of about one billion yen (about 6.7 million US dollars) would have been achieved. This large healthcare cost reduction for a single drug with an effective policy indicates the broader need for effective policies for other drugs, including other policies.

This study has several limitations. First, due to the characteristics of the database used, there is a bias present in the age and sex distribution of the subjects, which may not be generalisable to the entire Japanese population. Specifically, this database primarily covers employed individuals and their dependents under 75 years of age. The older demographic often has a higher burden of comorbidities and may exhibit different economic sensitivities or attitudes towards switching to biosimilar products. Consequently, they might have responded to the financial incentive policy assessed in this study differently to our younger study cohort, which may in turn limit the direct applicability of our findings to the entire rheumatoid arthritis population in Japan. Future studies which incorporate data on patients aged ≥75 years would provide valuable information on the policy's effectiveness across the full age spectrum of rheumatoid arthritis patients in Japan. Second, the data used in this study captured prescriptions up to March 2023, and any long-term trends beyond this period are not discernable. For infliximab, the relatively short duration of the post-intervention period may not have been sufficient to capture the long-term effects of the policy. This timeframe may not have been long enough to fully capture the long-term or delayed effects of the policy. This could lead to the policy's impact being underestimated or a more gradual onset of effect being undetected. Therefore, the null findings for infliximab must be interpreted with caution, and highlight the need for continued monitoring with extended follow-up data. Third, our study design was restricted by the absence of a suitable control group, which limits definitive causal attribution of observed changes to the reimbursement policy. Interrupted time series is a well-established quasi-experimental approach that effectively controls pre-existing secular trends, but alternative designs such as difference-in-differences could potentially have strengthened the causal inference. To partially address this concern, we examined the absolute monthly prescription counts for both originator and biosimilar products (Supplemental Figures S1A and S1B). For infliximab, total prescription volumes showed no abrupt shift around April 2020, when the etanercept incentive was introduced, suggesting that the etanercept findings are unlikely to be confounded by cross-drug substitution effects. Furthermore, we did not identify major treatment guideline revisions or significant biosimilar market events that coincided with either policy implementation date. Fourth, because our ITS analysis was conducted at the aggregate level, we were unable to incorporate individual-level determinants such as disease activity or severity, comorbidity burden, prior biologic treatment history, patient preference, or socioeconomic status (which is not reliably captured in the JMDC database). These factors are expected to remain relatively stable over time, however, and their influence on the observed trends is likely limited. Fifth, although individual-level switching between biologics cannot be completely ruled out, the overall stability of total infliximab volume during 2020–2021 (Supplemental Figure S1A) implies that any cross-drug migration was modest and therefore unlikely to significantly confound our main findings. Future studies using individual-level longitudinal data could quantify actual switching rates and new-start proportions, providing more granular insight into the behavioural mechanisms underlying the observed changes. Finally, the findings are based on specific reimbursement policies in Japan and might not directly apply to other countries with different healthcare systems, pricing regulations, or biosimilar markets. The external validity of this conclusion might be limited in the current situation wherein each country has its own biosimilar promotion policy. Nevertheless, we consider that the simplicity of Japan's policy likely represents a good case study for policy makers in the US and Europe.

## Conclusion

Our study demonstrates that the reimbursement incentive policy temporarily influenced the adoption of etanercept biosimilars in Japan, but provides only limited evidence for the effect on infliximab within 12 months post-implementation. These patterns highlight the complexity of the biosimilar market, and suggest that economic rewards alone will not simply increase penetration; rather, policies need to be dynamically adjusted to the characteristics of the individual drug and healthcare environment.

## Supplementary Material

Supplemental Material

## Data Availability

The authors are not permitted to share the data used in this study.
