# Supplementary material for: Financial reimbursement incentives in the use of biosimilars for rheumatoid arthritis in Japan
Source: J Pharm Policy Pract. 2026 Feb 25;19(1):2633832. doi: 10.1080/20523211.2026.2633832 (PMC12943821; doi:10.1080/20523211.2026.2633832)
Supplement: Supplemental Material [file JPPP_A_2633832_SM7132.docx]

eTable 1. Definitions of inclusion and exclusion criteria

| **Inclusion criteria** | **Definitions** |
| --- | --- |
| Infliximab | ATC code: L04AB02 |
| Originator | First 7 digits of YJ code: 2399402 |
| Biosimilar | First 7 digits of YJ code: 2399403, 2399404, 2399406 |
| Etanercept | ATC code: L04AB01 |
| Originator | First 7 digits of YJ code: 3999424 |
| Biosimilar | First 7 digits of YJ code: 3999448, 3999451 |
| Rheumatoid arthritis | ICD-10 code (standard disease code): M05, M060 (except 8844120), M062, M063, M068, M069 |
| **Exclusion criteria*** | **Definitions** |
| Behçet’s disease | ICD-10 code: M352 |
| Kawasaki disease | ICD-10 code: M303 |

Abbreviations: ATC, Anatomical Therapeutic Chemical; ICD-10, International Classification of Diseases, Tenth Revision; YJ, Yakka Joho.

*These criteria are exclusively applicable to the analysis of infliximab.

eTable 2. Sensitivity Analysis of Interrupted Time Series Model Estimates for Biosimilar Uptake with Varying Autocorrelation Adjustments

| **Monthly % change (95% CI)** |  | **Infliximab** | | **Etanercept** | |
| --- | --- | --- | --- | --- | --- |
| No Autocorrelation Adjustment | Level change | 0.14 | (−3.94, 4.22) | 13.48 | (9.26, 17.70) |
|  | Slope change | 0.21 | (−0.31, 0.73) | −1.09 | (−1.42, −0.76) |
| AR (1) Model  (Primary Model) | Level change | 0.14 | (−2.83, 3.11) | 13.48 | (7.82, 19.14) |
|  | Slope change | 0.21 | (−0.13, 0.55) | −1.09 | (−1.50, −0.68) |
| AR (2) Model | Level change | 0.14 | (−2.84, 3.13) | 13.48 | (6.83, 20.13) |
|  | Slope change | 0.21 | (−0.13, 0.55) | −1.09 | (−1.55, −0.64) |
| AR (3) Model | Level change | 0.14 | (−2.79, 3.07) | 13.48 | (6.13, 20.84) |
|  | Slope change | 0.21 | (−0.09, 0.51) | −1.09 | (−1.57, −0.62) |

Abbreviations: CI, confidence interval; AR (#), Autoregressive model of order # (e.g., AR (1) is a first-order autoregressive model).

(A)


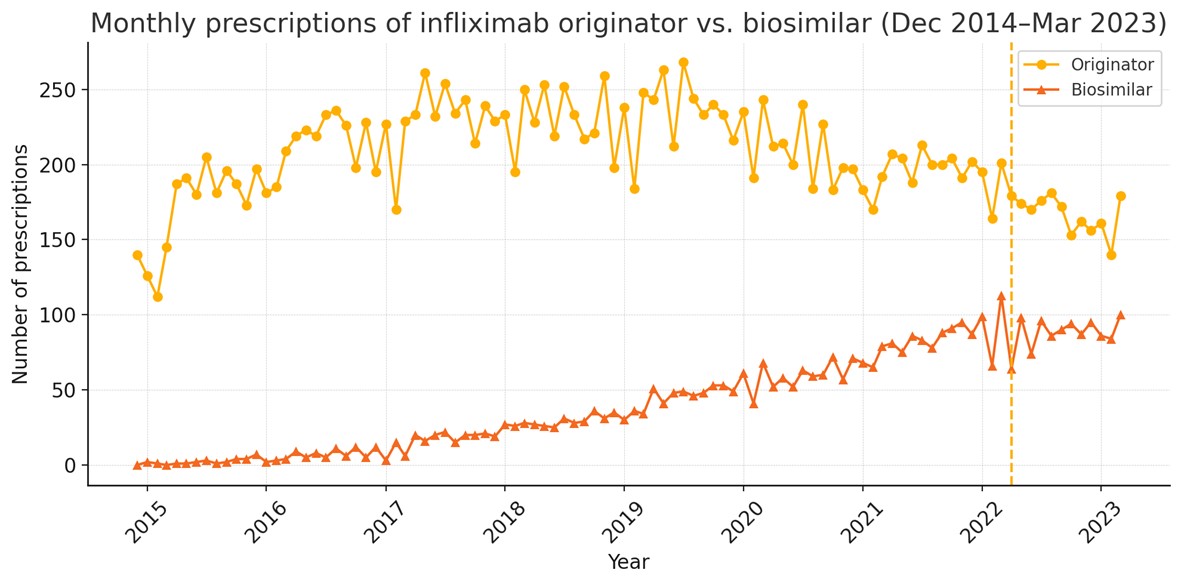


(B)


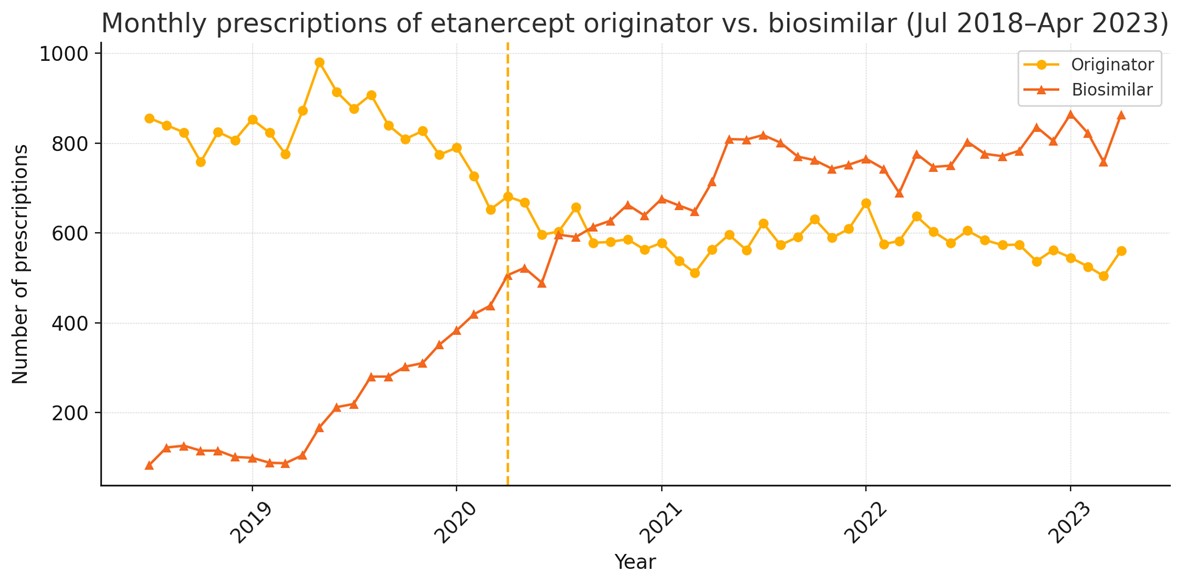


**Supplementary Figure S1.** Monthly prescription counts of originator and biosimilar products for infliximab (**A**) and etanercept (**B**).
